# Epidemic Erysipelas

**Published:** 1845-12-01

**Authors:** John G. House


### Epidemic Erysipelas.

Communicated for the Buffalo Medical Journal, by JOHN G. HOUSE, M. D.

SPRINGVILLE, Nov. 3, 1845.

Our village was visited last winter and spring, for the first time, by the epidemic sore throat and erysipelas which has so extensively prevailed in many parts of our country for the last four years. The disease, as it appeared here, was as evidently contagious as scarlatina or typhus. In one instance, out of seven females who were exposed to the miasm in their ministrations on the sick of one family, six were attacked with it in from twelve to seventy hours afterwards. There seems to be some trouble about a name for the epidemic; for whether it manifested itself in the throat, in the serous and mucous membranes, or on the skin, it was, most probably, one and the same disease, produced by the same cause. Neither the term *cynanche maligna*, nor erysipelas, is comprehensive enough to embrace the whole disease.

Those affected with the epidemic, were attacked with rigors, pain in the back of the head and neck, and in all the great nervous centres.—There were also rigidity and lameness of the muscles of the sides and back of the neck. In the most severe cases, the suffering was intolerable, manifested by groans and contortions. There were in many cases nausea and vomiting during the cold stage. The rigors were sooner or later succeeded by a reaction, corresponding in intensity with that of the chill. The posterior part of the fauces presented an erythematous, mahogany-red appearance, which, in the worst cases, was accompanied with very little tumefaction. This erythema manifested a disposition to metastasis: sometimes suddenly leaving the throat and attacking the skin, the peritoneum, the pleura or the mucous membrane of the bowels, or, else, extending itself to the larynx and trachea. Out of forty patients under my treatment, I lost two, and these by the last mentioned complication.—When the disease attacked those who had formerly had quinsy, or enlarged tonsils, these became much inflamed and tumified, inducing the patient to expect suppuration: but the swelling almost uniformly subsided without the formation of pus. The predisposition to superficial ulceration was so great as to overcome that to form abscess. When erysipelas occurred, it was of the deep-seated, phlegmonous variety, and was attended with less vesication than the ordinary sporadic disease. It frequently appeared within a few hours after the soreness of the fauces, upon the face, neck, genitals, or extremities: sometimes pain and soreness would commence in the hands or fingers where there happened to be the slightest abrasion of the cuticle, inflammation of the absorbents would speedily follow, and erysipelas would appear in the axilla. Adults of both sexes were more frequently subjects of the disease than children, but it must not be denied that the latter were often affected with scarlatina in the same families with the former. In fact, under modifying circumstances, the same epidemic constitution and contagion seemed capable of producing either cynanche, scarlatina, or puerperal peritonitis, and my friend Dr. Folts, has related to me the case of an adult female in which there was a full development of the eruption of scarlatina with erysipelas added—a strange compound. I might, if time and space allowed, have entered more minutely into the description of this epidemic, but let this suffice.

The first indication in the treatment was to overcome the cold stage as soon as possible, by the administration of Dover's Powder, warm, gently stimulating drinks, and external heat. As soon as reaction had taken place, we usually gave an emetic of Ipecacuanha, which uniformly mitigated the severity of the symptoms, relaxing spasm, and inducing more or less perspiration. Afterward, if necessary, we moved the bowels by means of the mildest laxatives, having found that full and frequent catharsis was unsafe and unnecessary. We then endeavored to keep up gentle diaphoresis by means of ipecac. and camphor, spts. of nitre, warm gruel, &c.

If these means failed to subdue the tension of pulse, we resorted to sanguineous depletion, graduating the amount by the strength of the patient, by the difference of frequency of the pulse in the lying or sitting posture, and by the color of the fauces. In all such cases, the benefit from moderate venesection was prompt and decisive, but in the two cases of laryngeal complication, this, with all other usual means, failed. One died from suffocation, and the other, after being given up to suffocate, was relieved of the cynanche by the spreading of erysipelas from a blister on the side of the neck. The patient, however, died three days afterward from exhaustion. As an application to the fauces, a solution of nitras argenti, six grains to the ounce, was found most serviceable in restoring tone to the disorganizing membrane, and staying the ulceration. This was applied twice daily, with a camels hair pencil. When the color of the fauces was very dark, we resorted to the common capsicum gargle with great advantage. An infusion of sage and alum was the best detergent. As a local application for erysipelas, we used the following: R. Potas. Nitrat. ʒ ii. Zinci sulph. ʒ i. Aqua pur. O i. Solve.

This, frequently applied to the surface, was as good as any thing, and what was no small consideration was its cheapness. In the latter stage of the disease, the patient required support from tonics, such as quinine combined with Dover's powder, gentian, columbo, &c. Convalescence was rather tedious, and sometimes rendered more so by abscesses under the fascia. The disease was quite prevalent from February to July, when it suddenly and almost entirely disappeared.

---

NOTE BY THE EDITOR.—We hope the foregoing communication will serve to call out others relating to Epidemic Erysipelas in different localities. It is very desirable in order to arrive at correct views of the pathology and therapeutics of this disease, to institute a comparison of the phenomena, and the effects of remedies, as observed in different places. Several contributions on the subject have appeared in other journals, and we should be glad to furnish our quota of facts for a comprehensive analysis, by which the character and best method of treatment, it is to be hoped, will be better understood than they are now.

So, also, as regards other diseases—nothing is better established than that various endemical influences, and those of a more general character which have been commonly referred, after Sydenham, to the atmosphere *(constitutio aeris)*, occasion the same diseases, at different times, and in different places, to assume widely different phases, and to require very different methods of treatment. A collection of facts, to serve as a basis for the scientific investigation of these variations, it is needless to say, is of very great importance. It can only be accomplished by the united efforts of different persons, located in different sections of country, who will feel sufficient interest in the object to make careful observations, and report them to the profession. To subserve this object is one of the most prominent of the functions of a medical journal. We are very desirous to render ours useful in this way; and shall be always glad to receive communications from those who are disposed to co-operate for this purpose.

